# Advanced Techniques for Liver Fibrosis Detection: Spectral Photoacoustic Imaging and Superpixel Photoacoustic Unmixing Analysis for Collagen Tracking

**DOI:** 10.3390/s24144617

**Published:** 2024-07-17

**Authors:** Laith R. Sultan, Valeria Grasso, Jithin Jose, Maryam Al-Hasani, Mrigendra B. Karmacharya, Chandra M. Sehgal

**Affiliations:** 1 Clinical Research Core, Department of Radiology, Children’s Hospital of Philadelphia, Philadelphia, PA 19104, USA; mbkarmacharya@gmail.com; 2 FUJIFILM VisualSonics, 1114 AB Amsterdam, The Netherlands; valeria.grasso@ucsf.edu (V.G.); jithin.jose@fujifilm.com (J.J.); 3 Department of Radiology, University of Pennsylvania, Philadelphia, PA 19104, USA; maryam.al-hasani@pennmedicine.upenn.edu (M.A.-H.); chandra.sehgal@pennmedicine.upenn.edu (C.M.S.)

**Keywords:** liver fibrosis, collagen, photoacoustics, multi-spectral imaging, molecular imaging, superpixel photoacoustic unmixing (SPAX)

## Abstract

Liver fibrosis, a major global health issue, is marked by excessive collagen deposition that impairs liver function. Noninvasive methods for the direct visualization of collagen content are crucial for the early detection and monitoring of fibrosis progression. This study investigates the potential of spectral photoacoustic imaging (sPAI) to monitor collagen development in liver fibrosis. Utilizing a novel data-driven superpixel photoacoustic unmixing (SPAX) framework, we aimed to distinguish collagen presence and evaluate its correlation with fibrosis progression. We employed an established diethylnitrosamine (DEN) model in rats to study liver fibrosis over various time points. Our results revealed a significant correlation between increased collagen photoacoustic signal intensity and advanced fibrosis stages. Collagen abundance maps displayed dynamic changes throughout fibrosis progression. These findings underscore the potential of sPAI for the noninvasive monitoring of collagen dynamics and fibrosis severity assessment. This research advances the development of noninvasive diagnostic tools and personalized management strategies for liver fibrosis.

## 1. Introduction

Liver fibrosis is a progressive condition with a significant global health burden [1,2]. It is characterized by excessive collagen deposition [3,4,5]. As liver fibrosis progresses, the balance between collagen production and degradation is disrupted, accumulating collagen fibers. Collagen type 3, particularly, forms a fibrous network in the liver, which results in the development of fibrotic scar tissue [6]. This scar tissue replaces the healthy liver tissue and impairs the liver’s ability to function properly. Collagen type 3 deposition is, consequently, related to more advanced stages of the disease. The early detection and monitoring of collagen development are, therefore, crucial for timely intervention and improved patient outcomes.

The current diagnostic methods for assessing collagen in liver fibrosis, involving liver biopsies and imaging techniques, have limitations. Liver biopsy, the gold standard for diagnosing liver fibrosis, is invasive and carries bleeding and infection risks [7]. A single biopsy contributes to sampling variability and does not fully represent the extent of fibrosis throughout the liver. Additionally, liver fibrosis is a dynamic disease that evolves with time, requiring repeated biopsy evaluations. Imaging techniques, such as ultrasound (US), computed tomography (CT), and magnetic resonance imaging (MRI), can provide a noninvasive assessment of liver fibrosis [8,9,10,11]. However, these methods indirectly infer fibrosis based on morphological and functional changes in the liver, which may reflect but not directly measure the collagen content [12]. Consequently, these techniques may have limited sensitivity and specificity in detecting early-stage fibrosis or accurately quantifying collagen content [13]. There continues to be a critical need for noninvasive methods that can directly visualize and quantify collagen content in liver fibrosis. The noninvasive assessment of tissue components such as collagen would provide a more accurate and reliable evaluation, enable the longitudinal monitoring of fibrosis progression, and facilitate early detection and intervention.

Photoacoustic imaging (PAI) is a novel noninvasive imaging method that uses the differences in optical absorption of chromophores within tissues to construct images [14,15,16]. PAI combines the high contrast of pure optical imaging and the deep penetration of US converted from light energy, to mitigate the influence of the strong light scattering of tissue [17,18]. This study explores the potential of PAI in monitoring collagen changes during liver fibrosis, shedding light on its emerging role in the noninvasive assessment and personalized management of this complex disease. The application of PAI for collagen quantification in fibrosis has been documented across various conditions [19,20,21,22,23]. For instance, Wang et al. employed PAI to measure collagen content in a mouse model of liver fibrosis, demonstrating a strong correlation between PAI signals and histologically assessed collagen levels [21]. Similarly, Zhu et al. utilized PAI to assess collagen deposition in pulmonary fibrosis, underscoring its ability to noninvasively monitor disease progression and evaluate therapeutic efficacy [22]. In another study, Zhang et al. investigated the use of PAI for quantifying collagen in skin fibrosis, highlighting its effectiveness in distinguishing fibrotic tissues from non-fibrotic ones [23]. Traditional PAI typically utilizes a single wavelength or a limited range of wavelengths, which can restrict its capability to differentiate between various tissue components. In contrast, spectral PAI employs multiple wavelengths, enhancing tissue characterization and providing more detailed and specific information about collagen content.

In the current study, we aim to assess the feasibility of spectral photoacoustic imaging (sPAI) to explore multi-spectroscopic absorption contrast for monitoring collagen changes during the progression of liver fibrosis. sPAI is a cutting-edge imaging technique that leverages the absorption properties of different tissue components at multiple wavelengths, enabling the detailed visualization and quantification of specific molecular features [24]. This method offers enhanced specificity and sensitivity in detecting fibrotic progression [25,26]. Recent advancements in sPAI have demonstrated its potential in capturing dynamic changes in collagen content and distribution within fibrotic tissues. For instance, Laufer et al. developed a method using multispectral photoacoustic tomography to quantify collagen in tissue phantoms, demonstrating the feasibility of this approach for in vivo applications [27]. Their technique utilized specific wavelengths to target collagen’s optical absorption characteristics, allowing for accurate detection and quantification. Similarly, Li et al. combined sPAI with US imaging to monitor collagen deposition in liver fibrosis, showing that this method could effectively track fibrosis progression with high spatial resolution [28]. However, these studies also have limitations, including the potential for spectral coloring and the need for accurate light fluence correction to avoid misinterpretations [29]. Spectral coloring, caused by variations in light absorption and scattering properties of different tissues, can lead to the inaccurate quantification of chromophores if not properly addressed. Additionally, light fluence correction is essential for accurate quantitative imaging, as variations in light penetration can significantly affect the photoacoustic signal [30].

A critical challenge with sPAI in differentiating tissue components is accurate multi-spectral unmixing. For this purpose, we employ a novel data-driven superpixel photoacoustic unmixing (SPAX) framework [31,32] to enable the differentiation of liver collagen composition with the progression of liver fibrosis. SPAX addresses many of the limitations mentioned earlier. SPAX combines spectral decoloring, automatic segmentation, and advanced unmixing algorithms to enhance the accuracy of collagen detection. Unlike traditional methods, SPAX does not require prior knowledge of the expected chromophore spectra, allowing for a fully automated and unbiased analysis [32]. The integration of superpixel subsampling improves the differentiation of both prominent and less prominent absorbers, thereby increasing the sensitivity and specificity of the analysis. One of the key advantages of the SPAX framework is its ability to perform spectral decoloring, which involves modeling the light fluence distribution to compensate for spectral coloring. This process ensures that the spectral unmixing is not biased by variations in light absorption and scattering, leading to the more accurate quantification of collagen and other chromophores. Moreover, the automatic segmentation feature of SPAX identifies tissue structures and delineates regions of interest, facilitating the precise analysis of collagen distribution and abundance.

By leveraging the strengths of SPAX, we aim to provide a more reliable and precise method for collagen quantification in liver fibrosis. The fully automated and data-driven nature of SPAX enhances the reproducibility and consistency of the results, making it a powerful tool for noninvasive diagnostics. This approach ultimately advances the development of noninvasive diagnostic tools and personalized management strategies for liver fibrosis. While our study primarily focuses on the collagen-specific information obtained from SPAX, we also included US radiomics measurements to offer additional insights into the structural changes associated with fibrosis. US imaging, widely used in clinical practice, provides valuable morphological and functional information that enhances fibrosis assessment. Our study utilizes a rat model of liver fibrosis induced by diethylnitrosamine (DEN) [33,34]. The DEN-induced rat model is well established for studying fibrosis, as it develops over time and closely mimics human inflammatory liver disease. In this study, we investigate the efficacy of sPAI and SPAX in detecting and quantifying collagen as fibrosis progresses in DEN rats over different time points. This provides a robust framework for future clinical applications.

## 2. Methods

### 2.1. Animal Liver Fibrosis Model

The University’s Institutional Animal Care and Use Committee approved all animal studies and protocols. The methods employed adhered to the applicable guidelines and regulations. Four male Wistar rats were obtained from Charles River Laboratories (Wilmington, MA, USA), weighing 350–400 g, and were acclimated in the housing facilities for one week. Following the acclimation period, the rats were given drinking water containing 0.01% diethylnitrosamine (DEN) from Sigma Aldrich, St. Louis, MO, USA, which they ingested freely for 12 weeks. After the imaging studies, the rats were euthanized using carbon dioxide asphyxiation. After euthanizing the animals, liver tissue samples were examined with trichrome staining for fibrotic changes. 

### 2.2. Histopathological Assessment

The necropsy samples from the liver lobes were preserved in 10% phosphate-buffered formalin for 48 to 72 h. They were then processed for histological examination using hematoxylin and eosin (H&E) and trichrome staining. Microscopic analysis of the slides was performed using an Olympus BX51 microscope (Olympus America Inc., Melville, NY, USA). Each histologic section was evaluated for hepatic fibrosis based on the METAVIR scoring system.

### 2.3. Photoacoustic Imaging Studies

sPAI signals were acquired from the liver in each animal using the Vevo LAZR photoacoustics imaging system (FUJIFILM VisualSonics, Toronto, ON, Canada) by Visualsonics. The sPAI imaging data were collected at various time points of DEN ingestion, including the baseline and at 5, 10, and 13 weeks later. A high-frequency (9–18 MHz) broadband transducer with axial and lateral resolutions of 100 μm and 235 μm, respectively, was used for the imaging. The imaging for sPAI were set within the wavelength range of 680 to 970 nm with presets as follows: PA Gain = 40 dB, wavelength range = 880–970 nm, and wavelength step increase = +5 nm. The imaging settings and time-gain compensation (TGC) remained constant for all the groups at different time points. 

During imaging, the rats were anesthetized using an inhalational isoflurane vaporizer (VetEquip Inc., Livermore, CA, USA) supplied with isoflurane vapor (2 to 4%) and oxygen gas (100 to 200 mL/min). Throughout the experiment, the rats were placed supine on the imaging platform, and a regulated amount of isoflurane and oxygen was continuously administered through a nose cone.

### 2.4. Spectral Photoacoustic Image Analysis Using Superpixel Multi-Component Unmixing (SPAX)

**Figure 1:**
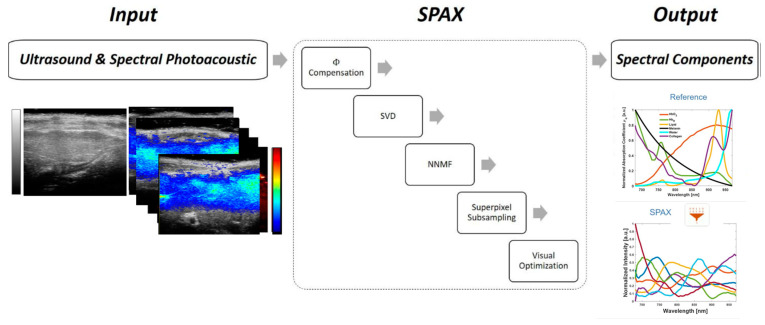
The workflow of the SPAX algorithm begins with the input of grayscale ultrasound and spectral photoacoustic imaging (sPAI) images, where the color bar represents the scale for photoacoustic signal intensity. The SPAX process includes multiple steps to correct signal intensity variations, SVD (Singular Value Decomposition) to reduce noise, NNMF (Non-Negative Matrix Factorization) to extract meaningful spectral components, superpixel subsampling for enhanced resolution, and visual optimization. The output displays spectral component curves for various tissue types, such as water, fat, and collagen, comparing reference spectra with SPAX-analyzed spectra.

To analyze the sPAI data, we employed the SPAX framework. SPAX is designed to enhance the accuracy of spectral unmixing by leveraging superpixel segmentation and advanced unmixing algorithms [31,32] (Figure 1). This framework is designed to process multi-spectral photoacoustic images within the wavelength range of 680 to 970 nm, which were co-registered with high-resolution ultrasound images (referred to as US-sPAI). The process begins with the delineation of the liver region of interest (ROI) in the sPAI datasets, which are co-registered with high-resolution ultrasound images. 

One of the primary advantages of the SPAX framework lies in its ability to automatically identify tissue spectral components without the need for manual intervention. This is achieved through a fully blind and automatic spectral unmixing process. Unlike traditional methods, SPAX does not require any prior knowledge or input of expected chromophore absorption spectra. Inherent to the SPAX algorithm is the incorporation of a superpixel subsampling approach. 

Key steps in the SPAX framework (Figure 1) include the following:

Spectral Decoloring: This step models the light fluence distribution to compensate for spectral coloring, which is caused by variations in light absorption and scattering properties of tissues [35]. US image segmentation and spectral Monte Carlo simulations based on a library of optical properties are used for this correction. Within the SPAX framework, an automated segmentation process is employed to identify the skinline, which serves as a watershed to distinguish tissue structures from the background. Following segmentation, fluence correction is performed to account for inhomogeneities caused by non-uniform light fluence distribution along the depth. This is critical for accurate spectral unmixing and for preventing misinterpretations due to inhomogeneous light fluence distribution.

Singular Value Decomposition (SVD): SVD is a mathematical technique that is utilized to enhance the robustness of the spectral unmixing process. It helps in automating the selection of hyperparameters and improving the efficiency of the algorithm. SVD decomposes the spectral data into orthogonal components, isolating the relevant signal from noise and artifacts [36,37]. The role of SVD in the SPAX framework is crucial for enhancing the spectral unmixing process. SVD facilitates the following:

Noise Reduction: by focusing on the largest singular values, SVD helps in filtering out the noise, retaining only the meaningful spectral information.

Hyperparameter Selection: SVD aids in the automatic selection of hyperparameters by identifying the most significant components, thereby reducing the need for manual tuning.

Robust Spectral Unmixing: The orthogonal basis vectors obtained from SVD provide a stable foundation for the unmixing algorithm, improving its robustness against variations in the data.

By incorporating SVD, the SPAX framework ensures a more accurate and efficient spectral unmixing process, enhancing the overall performance of collagen quantification in liver fibrosis.

Non-Negative Matrix Factorization (NNMF): This algorithm detects molecular components with different concentration values within the tissue [38,39,40,41]. It ensures a high sensitivity and specificity in identifying and quantifying the chromophores, such as collagen, within the liver [40]. NNMF decomposes the spectral data into two non-negative matrices, representing the spectral signatures of different tissue components and their spatial distributions. This non-negativity constraint ensures interpretability and facilitates the identification of collagen and other chromophores within liver tissues. By providing a part-based representation, NNMF aids in accurately distinguishing molecular components, crucial for monitoring fibrosis progression. Integrating NNMF with SPAX leverages advanced algorithms and automated segmentation techniques, improving the sensitivity and specificity of collagen detection. 

The quantification of molecular components is achieved through non-negative matrix factorization, providing concentration maps that indicate the relative abundance of collagen and other components. The concentration values are further validated by calculating correlation coefficients between the obtained spectra and reference spectra, such as collagen type 3. The quantification process involves the following metrics:

- Concentration Values: The SPAX framework provides concentration maps that indicate the relative abundance of collagen and other molecular components within the ROI.
- Correlation Coefficients: The similarity between the obtained spectra and reference spectra (e.g., collagen type 3) is quantified using correlation coefficients, providing a measure of how closely the detected components match the known spectra.

### 2.5. B-Mode US Quantitative Analysis

B-mode ultrasound (US) images of the liver were acquired at four time points during the progression of liver fibrosis in a rat model. The images were captured using the Visualsonics VevoLAZR system (Fujifilm, Toronto, ON, Canada). During the experiments, the rats were positioned supine under inhalational isoflurane vaporizer anesthesia (VetEquip Inc., Livermore, CA, USA) and oxygenated via a nose cone. Imaging presets were optimized and standardized with the following parameters: gain = 18 dB, high sensitivity, 100% power, transmit frequency of 21 MHz, and high line density.

An image analysis tool based on Interactive Data Language (IDL) [42,43] was used to extract first-order histogram features from these liver images at each time point. First-order statistics features included echo intensity (brightness level) and heterogeneity (local variance in gray levels within regions of interest). Echointensity and heterogeneity features were computed directly from the intensity of the pixels, derived from the mean intensity and standard deviation of intensity within the region of interest.

### 2.6. Data and Statistical Analysis

sPAI measured from the liverwas analyzed for each animal at baseline, 5 weeks, and 13 weeks following DEN diet initiation. sPAI signals from each rat were compared to a reference spectrum of collagen type 3 spectra obtained from the rat tail. The correlation coefficient between the reference and each animal spectra was measured to determine the similarity. All the statistical analyses were performed with MATLAB 2021b (Mathworks, Cambridge, MA, USA).

## 3. Results

**Figure 2:**
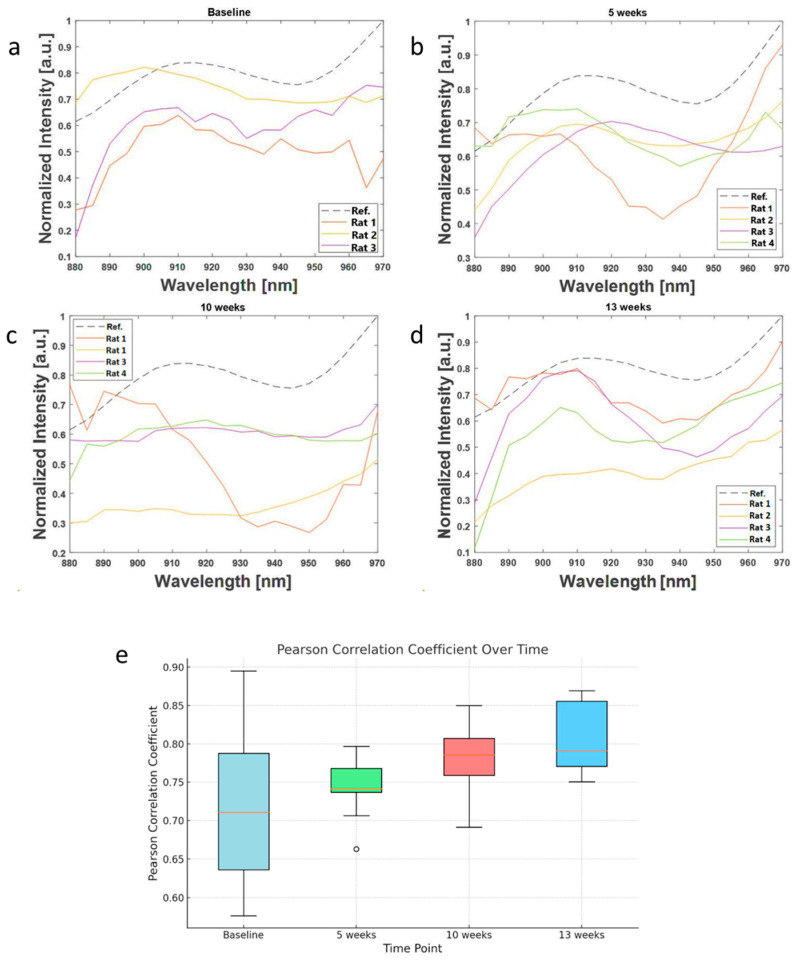
(**a**–**d**) Curves of photoacoustic signal intensity for each animal plotted and compared with a collagen reference spectrum curve (dotted line). (**e**) Box plot of correlation coefficients of sPAI spectrum for each animal with the collagen reference spectrum at each time point. The correlation is shown to be stronger towards advanced liver fibrosis stages particularly at 10 weeks and 13 weeks.

**The correlation of collagen photoacoustic signal with advanced fibrosis stages.** The sPAI signal intensity exhibited an increasing similarity to the collagen type 3 spectra as the disease advanced (Figure 2). In Figure 2a–c, the curves represent the sPAI spectra from each of the rats included in the study. These curves compared against the collagen reference spectrum at each time point visually demonstrated the increasing similarity to the reference spectrum as liver fibrosis progresses. The correlation coefficients for each time point quantify this similarity, providing a more objective measure of the degree of correlation between the sPAI signals and the collagen reference spectrum. The similarity between the two spectra was particularly evident at week 13. When we look at the correlation coefficient between the two curves, it increased with the progression of fibrosis (Figure 2b). The average correlation increased from 0.46 ± 0.22 at baseline to 0.6 ± 0.14, 0.6 ± 0.12, and 0.7 ± 0.09 for 5, 10, and 13 weeks of treatment, respectively. These results clearly illustrate the progression of liver fibrosis and the increasing presence of collagen type 3 in the liver tissue over time.

**Figure 3:**
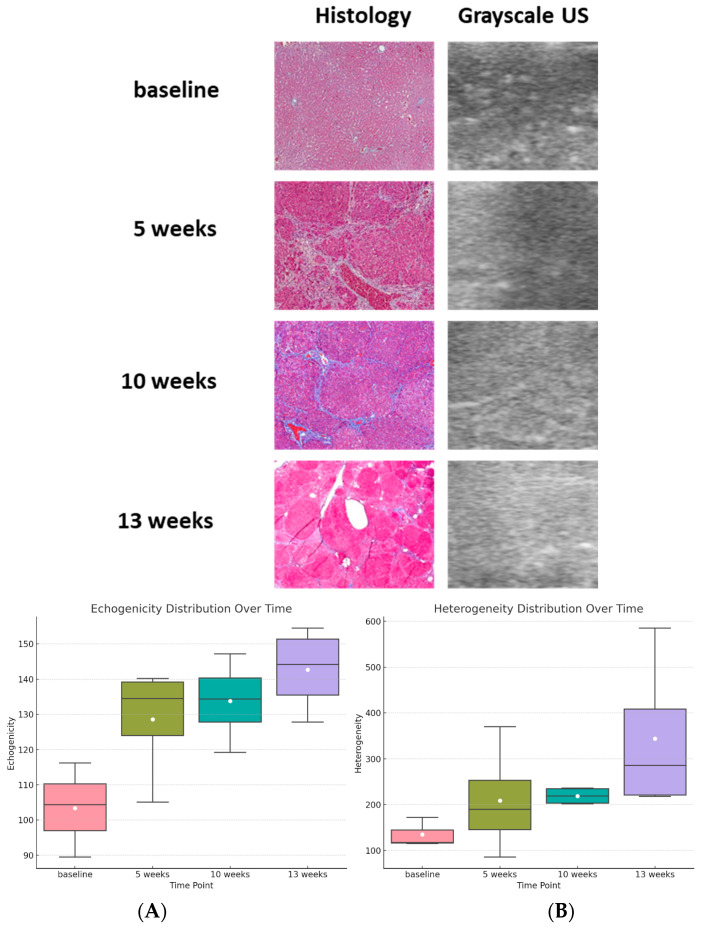
Histological and the corresponding grayscale ultrasound changes in liver fibrosis progression. The (**upper panel**) shows liver fibrosis progression assessed using trichrome staining for histological analysis. In parallel, B-mode grayscale ultrasound demonstrated a time-dependent increase in tissue echogenicity and heterogeneity, mirroring the histological changes. (**Lower panel**): the quantitative analysis of liver tissue properties using grayscale B-mode ultrasound imaging. (**A**) Echogenicity bar chart: shows a progressive increase in liver tissue echogenicity from baseline to 13 weeks of DEN treatment, reflecting increased tissue density due to fibrotic tissue accumulation. (**B**) Heterogeneity bar chart: demonstrates an increase in tissue heterogeneity over the same period, indicating greater variation in tissue texture and composition.

**Grayscale US findings correlate with sPAI changes in liver fibrosis.** The analysis of grayscale B-mode images reveals a consistent increase over time, which corresponds to the rise in PAI signal, as confirmed by histological findings (see Figure 3). On average, there is an incremental progression from the baseline values of 103.4 ± 13.4 and 135 ± 32.3 for echogenicity and heterogeneity, respectively. These values increase to 128.6 ± 16.3 and 208.9 ± 119.8 at five weeks, further progressing to 133.7 ± 11.8 and 218.8 ± 18.8 at ten weeks. The most significant increase is observed at the 13-week mark, with values reaching 142.7 ± 12.1 for echogenicity and 343.8 ± 172.1 for heterogeneity. In Figure 3, the upper panel of displays histological sections of liver tissue stained with trichrome at each time point, versus the corresponding B-mode US images, revealing the progression, which is visually apparent, with more structural alterations correlating with advanced fibrosis stages. The lower panel includes two bar charts that quantify changes in liver tissue properties as observed through grayscale B-mode ultrasound imaging. The first bar chart illustrates a progressive increase in echogenicity, reflecting increased tissue density due to fibrotic tissue accumulation. The second bar chart shows an increase in tissue heterogeneity, indicating greater variation in tissue texture and composition. By comparing these metrics, we gain a comprehensive understanding of the structural and compositional changes within the liver during fibrosis progression.

**Figure 4:**
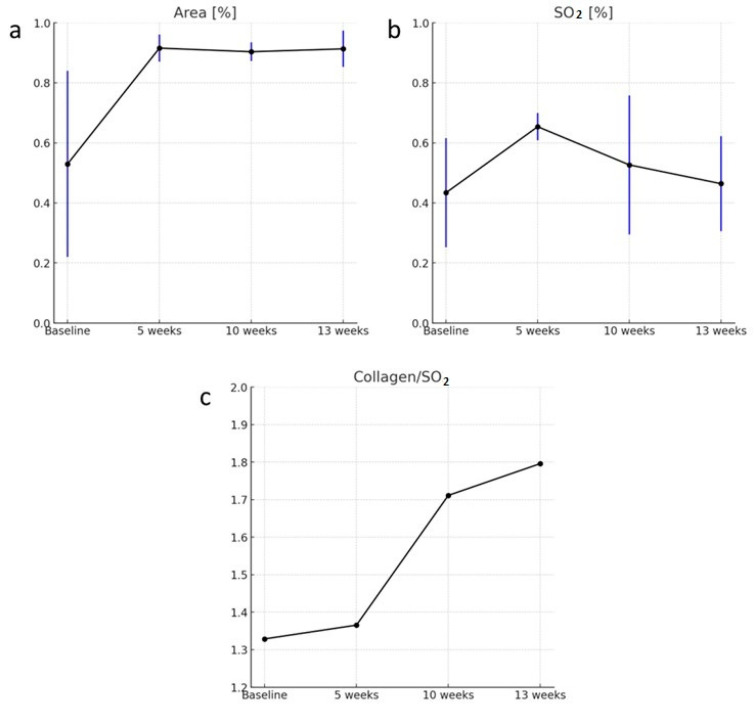
This figure shows (**a**) mean percentage area of PAI signals changes over time, with a clear increase from baseline to 5 weeks, then surprisingly, it goes down at 10 weeks but rises again at 13 weeks. (**b**) shows oxygen saturation (SO_2)_ changes over time. The blue lines represent standard deviation. (**c**) Curve of ratio of percentage PAI area of collagen/percent SO_2_. The normalized value, and after taking the area occupied by O_2_ into consideration, the curve shows an increase from baseline to 5 weeks but shows a drastic change toward 10 and 13 weeks.

**Abundance maps of collagen showed more increase with liver fibrosis progression.** We evaluated the change in percentage area of the sPAI signal over time. The abundance of collagen within the liver lobe region was measured and compared across the different time points. The area was defined based on the regions of interest (ROIs) delineating the liver tissue images using the SPAX framework. The results revealed interesting trends (Figure 4a). From the baseline to the 5-week time point, there was an increase in the percentage area, indicating a higher abundance of collagen within the liver. However, surprisingly, at the 10-week time point, there was a decrease in the percentage area, suggesting a reduction in collagen abundance. Nevertheless, at the 13-week time point, the percentage area increased once again, indicating an elevated presence of collagen within the liver. When we evaluated the ratio of collagen per unit of tissue oxygen saturation (SO_2_) (Figure 4b,c). We found a trend of increased collagen content with disease progression, suggesting a potential association between fibrosis development and alterations in collagen abundance after leaving out the tissue oxygenation effect.

## 4. Discussion

The deposition of collagen, specifically collagen type 3, in liver fibrosis is associated with the ongoing damage, scarring, and ultimate disruption of the liver structure and function [3,4,5,6]. Early detection, therefore, can help manage and reduce collagen deposition and address the underlying causes of liver fibrosis to preserve liver function and prevent further complications. The present study aims to evaluate sPAI as a novel noninvasive imaging tool to monitor the dynamics of collagen accumulation within the liver during fibrosis progression. The study findings demonstrate the feasibility of measuring changes in collagen content, as well as its relationship with disease progression.

Collagen is commonly a weaker absorber within the NIR-I range of 680–970 nm. Thus, it is challenging to be automatically unmixed. To overcome this limitation, we utilized the SPAX framework that includes a combination of procedures to overcome the current limitations such as supervised unmixing techniques and misinterpretations caused by spectral coloring. The framework also compensates for the changes in the spectral shape along depth due to light fluence variations. Thus, the SPAX framework would be beneficial and open many possibilities to monitor molecular changes. SPAX can detect molecular tissue components and their volumetric distribution from spectral photoacoustic imaging. Our previous research demonstrated that the SPAX approach exhibits high sensitivity to spectral unmixing, making it capable of detecting spectral changes that may occur in the disease process [21]. Studies have validated that the SPAX framework is more sensitive than traditional blind source separation (BSS) methods when applied to sPAI [23]. SPAX shows greater robustness against nonlinearities from fluence estimation inaccuracies, enabling more efficient convergence on source components with the help of a positivity constraint. In poorly conditioned scenarios, such as low SNR, SPAX outperforms traditional methods, providing more accurate results. This advantage comes from SPAX’s part-based decomposition, which breaks the signal into meaningful components, enhancing information extraction even in challenging conditions.

In the current study, we employed unmixed sPAI to analyze the signal intensity between 880 and 970 nm in the liver at different time points, aiming to assess the correlation between the obtained spectra and a reference point derived from collagen 3 spectra taken from the rat tail. The wavelengths between 880 and 970 nm are used for collagen detection based on the absorption properties of collagen fibers in this range [24,25,26]. Collagen has a strong absorption peak in the near-infrared region, particularly around 930 nm. Therefore, by using wavelengths within this range, sPAI technique can effectively generate signals from collagen as the maximum absorption peak of collagen can be targeted. In addition, the absorption of other components in this range such as blood and water is relatively low [24], and this reduce this potential interference from these components, enabling more collagen detection. An advantage of using this sPAI wavelength range is that near-infrared light can penetrate deeper into biological tissues, and this allows the detection of collagen in deeper tissues, making it suitable for applications in biomedical imaging.

The analysis involved calculating the correlation coefficient between the liver spectra and the reference collagen type 3 spectra obtained from rat tail [44]. Collagen from rat tails was used to provide a biologically relevant context that closely aligns with the experimental conditions of interest. The structural complexity and interactions of collagen within rat tail tissues may more accurately mirror the in vivo environment than collagen obtained from commercial sources, which is typically isolated from bovine or porcine skin and may undergo extensive processing [45]. Utilizing rat tail collagen ensures that the biochemical properties and tissue interactions studied are representative of those in the species under investigation. Moreover, sourcing collagen from the same species significantly reduces biological variability, thus enhancing the reproducibility and relevance of our findings. This approach ensures that the experimental conditions are consistent and that the results are directly applicable to the physiological contexts being modeled. The results revealed interesting insights, with average correlation coefficient increasing as the disease progressed over time. This suggests that the presence of collagen 3 is closely associated with the progression of liver fibrosis and, as the disease advanced, there was a stronger association between the liver spectra and collagen 3. This finding is particularly notable at the 13-week time point, where a very strong correlation coefficient was observed, indicating a significant presence of collagen 3 and a higher degree of fibrosis. This finding aligns with the well-established role of collagen in fibrotic processes, where excessive collagen deposition leads to tissue remodeling and scarring. The ability to quantitatively assess the correlation between liver spectra and collagen 3 using PAI has important clinical implications. It provides a noninvasive means to monitor and assess the progression of liver fibrosis. The increasing correlation coefficient indicates a higher likelihood of fibrotic changes within the liver, which can be a valuable marker for disease severity and progression.

The assessment of the percentage area of the PAI signal, indicative of collagen abundance, revealed dynamic changes over time. An initial increase in the collagen percentage area from baseline to the 5-week mark suggests early fibrosis development, consistent with prior studies identifying collagen accumulation as a hallmark of fibrotic processes. However, the unexpected decrease in collagen percentage area at the 10-week point raises intriguing questions about the underlying mechanisms and transitional stages of fibrosis progression. Further investigation is needed to elucidate the factors contributing to this decrease. Notably, when we normalized collagen levels to O_2_ by calculating the ratio to reduce the effect of oxygen variability, we observed a clear trend of increasing collagen levels over the 13-week period. This normalization provides a clearer perspective on collagen dynamics without the confounding influence of oxygen level fluctuations. The evaluation of the collagen per unit of tissue oxygen saturation (SO_2_) ratio offered insights into the relationship between collagen content and tissue oxygenation. By considering the oxygenation levels, we can more accurately assess collagen abundance relative to the physiological state of the tissue. In addition, a higher collagen to SO_2_ ratio suggests increased collagen deposition in regions with lower oxygenation, which is indicative of fibrotic tissue. This provides insights into the relationship between fibrosis progression and tissue oxygenation, highlighting areas where fibrosis may be more advanced. The increasing trend in the collagen per SO_2_ unit ratio suggests that fibrotic tissue may experience compromised oxygen supply and reduced perfusion. This finding is in line with previous studies demonstrating altered microvascular networks and oxygen delivery in fibrotic organs [46,47]. The link between collagen accumulation and tissue oxygenation adds a crucial dimension to our understanding of fibrosis and its pathophysiology.

The inclusion of US radiomics analysis in our study provides complementary information to sPAI. While sPAI offers high sensitivity to molecular changes, such as collagen content, US imaging captures morphological changes and tissue heterogeneity, which are also indicative of fibrosis progression. By comparing histological results with US imaging, we aim to validate the structural changes observed in PAI and provide additional quantitative metrics for fibrosis evaluation. This multimodal approach enhances the overall assessment of liver fibrosis and underscores the potential of combining sPAI and ultrasound for noninvasive diagnostics. While sPAI offers molecular insights and is primarily used in our study to quantify collagen content, our goal was to demonstrate how sPAI and ultrasound together offer a comprehensive view of fibrosis progression, rather than solely focusing on molecular vs. histological comparison. We acknowledge the importance of direct comparisons between sPAI and histological results and plan to address this in future studies, where we aim to develop standardized protocols for such comparisons.

While these findings provide valuable insights into the dynamics of collagen changes during fibrosis progression, few limitations should be acknowledged. The study was conducted using a small animal group; therefore, further validation in human subjects is necessary to confirm the translational relevance. The mechanisms underlying the observed changes in collagen and its relationship with disease progression require further investigation in the human model. In addition, challenges related to standardization and depth limitations are needed for further validation through clinical trials. While the longitudinal design and repeated measures across different time points provide a robust dataset for analysis, we agree that further studies with larger sample sizes and additional validation in other models are necessary to confirm our findings and enhance their generalizability.

In conclusion, sPAI has a unique ability to detect and differentiate collagen based on its strong optical absorption properties. We observed that, at specific wavelengths, we can selectively target collagen, allowing for the visualization and quantification of collagen-rich areas within the liver. The study findings have implications for developing noninvasive diagnostic tools, monitoring disease progression, and identifying potential therapeutic targets in liver fibrosis. Further research is warranted to unravel the underlying mechanisms and validate these findings in clinical settings and to comprehend the potential clinical impact of photoacoustic imaging in liver fibrosis monitoring.

## Data Availability

The data presented in this study are available on request from the corresponding author.

## References

[1] Asrani S.K., Devarbhavi H., Eaton J., Kamath P.S. (2019). Burden of liver diseases in the world. J. Hepatol..

[2] Roehlen N., Crouchet E., Baumert T.F. (2020). Liver Fibrosis: Mechanistic Concepts and Therapeutic Perspectives. Cells.

[3] Bataller R., Brenner D.A. (2005). Liver fibrosis. J. Clin. Investig..

[4] Liu T., Wang X., Karsdal M.A., Leeming D.J., Genovese F. (2012). Molecular Serum Markers of Liver Fibrosis. Biomark. Insights.

[5] Rojkind M., Giambrone M.-A., Biempica L. (1979). Collagen Types in Normal and Cirrhotic Liver. Gastroenterology.

[6] Karsdal M.A., Henriksen K., Nielsen M.J., Byrjalsen I., Leeming D.J., Gardner S., Goodman Z., Patel K., Krag A., Christiansen C. (2016). Fibrogenesis assessed by serological type III collagen formation identifies patients with progressive liver fibrosis and responders to a potential antifibrotic therapy. Am. J. Physiol. Gastrointest. Liver Physiol..

[7] Heyens L.J.M., Busschots D., Koek G.H., Robaeys G., Francque S. (2021). Liver Fibrosis in Non-alcoholic Fatty Liver Disease: From Liver Biopsy to Noninvasive Biomarkers in Diagnosis and Treatment. Front. Med..

[8] D’Souza J.C., Sultan L.R., Hunt S.J., Schultz S.M., Brice A.K., Wood A.K., Sehgal C.M. (2019). B-mode ultrasound for the assessment of hepatic fibrosis: A quantitative multiparametric analysis for a radiomics approach. Sci. Rep..

[9] Gerstenmaier J.F., Gibson R.N. (2014). Ultrasound in chronic liver disease. Insights Imaging.

[10] Li S., Sun X., Chen M., Ying Z., Wan Y., Pi L., Ren B., Cao Q. (2019). Liver Fibrosis Conventional and Molecular Imaging Diagnosis Update. J. Liver..

[11] Rix A., Lederle W., Theek B., Lammers T., Moonen C., Schmitz G., Kiessling F. (2018). Advanced Ultrasound Technologies for Diagnosis and Therapy. J. Nucl. Med..

[12] Zhang Y.N., Fowler K.J., Hamilton G., Cui J.Y., Sy E.Z., Balanay M., Hooker J.C., Szeverenyi N., Sirlin C.B. (2018). Liver fat imaging-a clinical overview of ultrasound, CT, and MR imaging. Br. J. Radiol..

[13] Salarian M., Turaga R.C., Xue S., Nezafati M., Hekmatyar K., Qiao J., Zhang Y., Tan S., Ibhagui O.Y., Hai Y. (2019). Early detection and staging of chronic liver diseases with a protein MRI contrast agent. Nat. Commun..

[14] Lv J., Xu Y., Xu L., Nie L. (2021). Quantitative Functional Evaluation of Liver Fibrosis in Mice with Dynamic Contrast-enhanced Photoacoustic Imaging. Radiology.

[15] Li W., Chen R., Lv J., Wang H., Liu Y., Peng Y., Qian Z., Fu G., Nie L. (2017). In vivo photoacoustic imaging of brain injury and rehabilitation by high-efficient near-infrared dye labeled mesenchymal stem cells with enhanced brain barrier permeability. Adv. Sci..

[16] Liu Y., Nie L., Chen X. (2016). Photoacoustic molecular imaging: From multiscale biomedical applications towards early-stage theranostics. Trends Biotechnol..

[17] Bell K., Hajireza P., Zemp R. (2018). Scattering cross-sectional modulation in photoacoustic remote sensing microscopy. Opt. Lett..

[18] Lv J., Peng Y., Li S., Guo Z., Zhao Q., Zhang X., Nie L. (2018). Hemispherical photoacoustic imaging of myocardial infarction: In vivo detection and monitoring. Eur. Radiol..

[19] Hysi E., He X., Fadhel M.N., Zhang T., Krizova A., Ordon M., Farcas M., Pace K.T., Mintsopoulos V., Lee W.L. (2020). Photoacoustic imaging of kidney fibrosis for assessing pretransplant organ quality. JCI Insight.

[20] Huang G., Lv J., He Y., Yang J., Zeng L., Nie L. (2022). In vivo quantitative photoacoustic evaluation of the liver and kidney pathology in tyrosinemia. Photoacoustics.

[21] Wang L.V., Hu S. (2017). Photoacoustic tomography: In vivo imaging from organelles to organs. Science.

[22] Zhu Y., Wang L.V. (2019). Photoacoustic imaging and characterization of the microvasculature. J. Biomed. Opt..

[23] Zhang E., Laufer J. (2021). Photoacoustic imaging of skin: In vivo measurement of epidermal thickness and blood flow. J. Biomed. Opt..

[24] Lee H., Kim J., Kim H.H., Kim C.S., Kim J. (2022). Review on Optical Imaging Techniques for Multispectral Analysis of Nanomaterials. Nanotheranostics.

[25] Wang L., Gao L. (2020). Spectral photoacoustic imaging for dynamic monitoring of liver fibrosis. Biomed. Opt. Express.

[26] Zhang X., Li C. (2021). Longitudinal monitoring of liver fibrosis progression and therapeutic response using spectral photoacoustic imaging. J. Hepatol..

[27] Laufer J., Delpy D., Elwell C., Beard P. (2010). Quantitative spatially resolved measurement of tissue chromophore concentrations using photoacoustic spectroscopy: Application to the measurement of blood oxygenation and hemoglobin concentration. Phys. Med. Biol..

[28] Li Y., Wang P., Zhu L., Zhou Y. (2018). Quantitative imaging of tissue optical absorption properties using photoacoustic imaging. J. Biomed. Opt..

[29] Wang X., Xie X., Ku G., Wang L.V., Stoica G. (2012). Noninvasive imaging of hemoglobin concentration and oxygenation in the rat brain using high-resolution photoacoustic tomography. J. Biomed. Opt..

[30] Tzoumas S., Nunes A., Olefir I., Stangl S., Symvoulidis P., Glasl S., Bayer C., Multhoff G., Ntziachristos V. (2016). Eigenspectra optoacoustic tomography achieves quantitative blood oxygenation imaging deep in tissues. Nat. Commun..

[31] Grasso V., Willumeit-Römer R., Jose J. (2022). Superpixel spectral unmixing framework for the volumetric assessment of tissue chromophores: A photoacoustic data-driven approach. Photoacoustics.

[32] Grasso V., Hassan H.W., Mirtaheri P., Willumeit-Römer R., Jose J. (2022). Recent advances in photoacoustic blind source spectral unmixing approaches and the enhanced detection of endogenous tissue chromophores. Front. Sig. Proc..

[33] Sultan L.R., Karmacharya M.B., Hunt S.J., Wood A.K.W., Sehgal C.M. (2021). Subsequent Ultrasound Vascular Targeting Therapy of Hepatocellular Carcinoma Improves the Treatment Efficacy. Biology.

[34] D’Souza J.C., Sultan L.R., Hunt S.J., Gade T.P., Karmacharya M.B., Schultz S.M., Brice A.K., Wood A.K.W., Sehgal C.M. (2019). Microbubble-enhanced ultrasound for the antivascular treatment and monitoring of hepatocellular carcinoma. Nanotheranostics.

[35] Gröhl J., Kirchner T., Adler T.J., Hacker L., Holzwarth N., Hernández-Aguilera A., Herrera M.A., Santos E., Bohndiek S.E., Maier-Hein L. (2021). Learned spectral decoloring enables photoacoustic oximetry. Sci. Rep..

[36] Padua D. (2011). Singular-Value Decomposition (SVD). Encyclopedia of Parallel Computing.

[37] Schmidt M., Rajagopal S., Ren Z., Moffat K. (2003). Application of singular value decomposition to the analysis of time-resolved macromolecular x-ray data. Biophys. J..

[38] Carbonero D., Noueihed J., Kramer M.A., White J.A. (2024). Non-Negative Matrix Factorization for Analyzing State Dependent Neuronal Network Dynamics in Calcium Recordings. bioRxiv.

[39] Lee D., Seung H. (1999). Learning the parts of objects by non-negative matrix factorization. Nature.

[40] Smith R.T., Jones A.B. (2020). Application of Non-negative Matrix Factorization in Biomedical Imaging. J. Biomed. Opt..

[41] Devarajan K. (2008). Nonnegative matrix factorization: An analytical and interpretive tool in computational biology. PLoS Comput. Biol..

[42] Al-Hasani M., Sultan L.R., Sagreiya H., Cary T.W., Karmacharya M.B., Sehgal C.M. (2022). Ultrasound Radiomics for the Detection of Early-Stage Liver Fibrosis. Diagnostics.

[43] Sultan L.R., Cary T.W., Al-Hasani M., Karmacharya M.B., Venkatesh S.S., Assenmacher C.-A., Radaelli E., Sehgal C.M. (2022). Can Sequential Images from the Same Object Be Used for Training Machine Learning Models? A Case Study for Detecting Liver Disease by Ultrasound Radiomics. AI.

[44] Sekar S.K., Bargigia I., Mora A.D., Taroni P., Ruggeri A., Tosi A., Pifferi A., Farina A. (2017). Diffuse optical characterization of collagen absorption from 500 to 1700 nm. J. Biomed. Opt..

[45] Karsdal M.A., Daniels S.J., Holm Nielsen S., Bager C., Rasmussen D.G.K., Loomba R., Surabattula R., Villesen I.F., Luo Y., Shevell D. (2020). Collagen biology and non-invasive biomarkers of liver fibrosis. Liver Int..

[46] Karmacharya M.B., Sultan L.R., Kirkham B.M., Brice A.K., Wood A.K.W., Sehgal C.M. (2020). Photoacoustic Imaging for Assessing Tissue Oxygenation Changes in Rat Hepatic Fibrosis. Diagnostics.

[47] Sultan L., Karmacharya M., Kirkham B., D’Souza J., Hunt S.J., Brice A., Wood A.K., Sehgal C. A Hybrid Quantitative Ultrasound and Photoacoustic Imaging Approach for Detection and Monitoring of Liver Fibrosis. Proceedings of the the Liver Meeting Digital Experience™, AASLD.

